# Effects of Vitamin D3 Treatment on Polycystic Ovary Symptoms: A Prospective Double-Blind Two-Phase Randomized Controlled Clinical Trial

**DOI:** 10.3390/nu17071246

**Published:** 2025-04-02

**Authors:** Béla E. Tóth, István Takács, Zsuzsanna Valkusz, Attila Jakab, Zsanett Fülöp, Kristóf Kádár, Zsuzsanna Putz, János Pál Kósa, Péter Lakatos

**Affiliations:** 1 Department of Pharmaceutical Surveillance and Economy, Faculty of Pharmacy, University of Debrecen, 4032 Debrecen, Hungary; fulopzsanetta@gmail.com; 2 Department of Internal Medicine and Oncology, Semmelweis University, 1083 Budapest, Hungary; takacs.istvan@semmelweis.hu (I.T.); zsuzsannaputz@yahoo.com (Z.P.); kosa.janos@semmelweis.hu (J.P.K.); lakatos.peter@semmelweis.hu (P.L.); 3 Department of Internal Medicine, Faculty of Medicine, University of Szeged, 6725 Szeged, Hungary; valkusz.zsuzsanna@med.u-szeged.hu; 4 Department of Obstetrics and Gynaecology, Faculty of Medicine, University of Debrecen, 4032 Debrecen, Hungary; ja@med.unideb.hu; 5 Department of Oral Biology, Semmelweis University, 1089 Budapest, Hungary; kadkris@outlook.hu

**Keywords:** vitamin D3, PCOS, ovarian morphology, ovulatory dysfunction, LH/FSH, progesterone, testosterone, 25(OH)D, cycle length

## Abstract

Background/Objectives: Vitamin D deficiency is common in women with polycystic ovary syndrome (PCOS) and may be associated with metabolic and endocrine disorders as well as ovulatory dysfunction. Vitamin D supplementation may improve ovarian dysfunction and follicular development by effecting gene expression. The aim of the present study was to investigate the effects of vitamin D supplementation in women with PCOS through a prospective, randomized, two-phase, parallel design, placebo-controlled trial. Methods: We assessed the impact on ovarian morphology, cycle length, and ovulatory dysfunction. Transvaginal ultrasonography (TVUS) examinations and clinical laboratory assessments were conducted at the baseline, and again after 12 and 24 weeks. The participants received vitamin D (30,000 IU/week) or a placebo (without concurrent metformin use) for 12 weeks, supplemented with calcium, followed by an additional 12 weeks of vitamin D treatment. Results: The treatment resulted in improvements in ovarian morphology and regularity of menstrual cycles in more than half of the patients. Additionally, vitamin D3 was associated with a significant increase in the ovulation rate. A statistically significant reduction in mean testosterone levels was observed in the subgroup of patients with an LH/FSH ratio greater than 2. Conclusions: Our results suggest that vitamin D3 treatment could function as either a standalone or an adjunctive therapy in the management of PCOS.

## 1. Introduction

A significant number of clinical and experimental data have already demonstrated that in addition to classical skeletal actions and the regulation of calcium homeostasis, vitamin D might have a regulatory role in several polycystic ovary syndrome (PCOS)-associated symptoms of the reproductive system, including ovulatory dysfunction, hyperandrogenism as well as insulin resistance and metabolic dysfunction in women [1,2,3,4,5,6]. The results of observational studies revealed the association between a low vitamin D status and PCOS [3,7,8,9]. Epidemiological studies revealed the relatively high prevalence of vitamin D deficiency in PCOS in comparison with the general population [10]. Among the clinical manifestations of PCOS with androgen excess, a higher prevalence of vitamin D deficiency was found [11]. Vitamin D participates in the regulation of sex hormones and through the expression of vitamin D receptor (VDR) and 1α-hydroxylase activity; it can also affect the activity of reproductive tissues, such as the ovaries [12], uterus [13,14], placenta [15], pituitary [16], and hypothalamus. Several studies have examined the potential effects of vitamin D on ovarian function [1,17].

Data from interventional clinical trials in PCOS women intended to demonstrate a causal link between vitamin D levels and PCOS concluded that even though vitamin D might have an impact on PCOS [7,18,19,20], the treatment may not significantly improve the metabolic and endocrine parameters considered as markers of PCOS. The results of these previous studies investigating the effects of vitamin D supplementation on PCOS were controversial. However, repleted vitamin D levels might revert effectively the regularity of menstrual cycles. The probability of ovulation was found to be correlated with vitamin D levels in PCOS [21]. On the other hand, obesity, metabolic disorders, and insulin resistance are common among PCOS patients and hypothesized to be related to impaired ovarian function.

Vitamin D deficiency has also been shown to be associated with impaired glucose clearance and insulin secretion. The hypothesis has been tested in several clinical trials with vitamin D supplementation alone [4,20,22,23,24] or in combination with metformin [5,25,26,27,28,29,30]. Vitamin D improves glucose metabolism which was suggested as one of the putative connections [31,32,33].

Vitamin D deficiency has been reported to contribute to the pathogenesis of several chronic conditions including endocrine diseases [34,35,36,37,38,39]. The expression of VDR in ovarian cells suggests that vitamin D has a role in the steroidogenesis of females by modulating the activity of key enzymes involved in this process [17,32,40,41]. It may also influence the androgen/estrogen balance. It was concluded that the vitamin D pathway might have a regulatory role in several PCOS-associated symptoms, including menstrual irregularities, ovulatory dysfunction, and endocrine alterations. PCOS is considered as a multifactorial disorder where endocrine, genetic, and environmental factors play a role, but the root cause has not yet been established. Vitamin D supplementation (in combination with calcium) could be a simple, cost-effective add-on therapy with high benefits/risk ratio; however, the results of meta-analyses based on randomized controlled trials (RCTs) is still showing some controversy in regard to the outcomes [42,43,44].

The aim of the present study was to investigate the effect of 30,000 IU/week vitamin D supplementation in PCOS in a prospective randomized two-phase parallel design, including placebo-controlled lead-in and open-label-extended supplementation phases. The selected dosing scheme was based on our prior findings supporting the effectiveness and safety of 30,000 IU/week in vitamin D deficient patients [45,46]. That two-phase study design allowed us to investigate the effect of vitamin D supplementation in vitamin D-depleted patients, as well as for sufficiently repleted patients. Our primary goal was to assess the effect of vitamin D supplementation (with no concomitant use of metformin) on the ovarian morphology and cycle dysfunction. An additional post-hoc analysis investigated a hyperandrogenic subgroup, as well as the trial subjects with a higher LH/FSH ratio in regard to the responsiveness to vitamin D repletion.

## 2. Materials and Methods

### 2.1. Study Design

**Figure 1:**
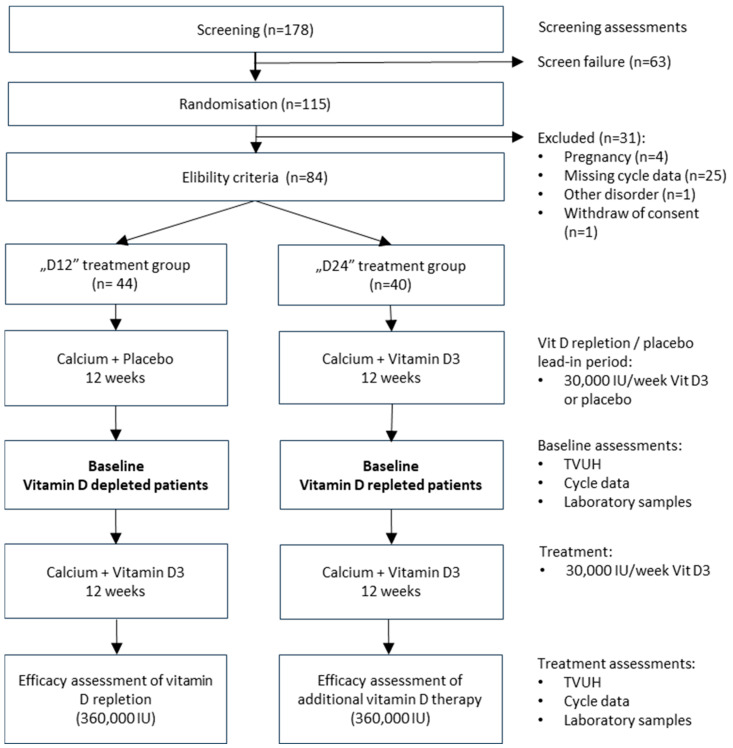
Study flow chart and schedule of assessments.

This study was a prospective multicentre, randomized, two-phase, double-blind, placebo-controlled, parallel design trial conducted at 8 clinical study sites in Hungary between 2016 and 2020. The trial was intended to investigate the effects of the weekly administration of vitamin D supplementation or a placebo over 12 weeks followed by a 12-week-long open-label period to assess the ovarian function in women diagnosed with PCOS. The treatment group “D12” received a placebo for 12 weeks first and then 360,000 IU for 12 weeks. Patients in the “D24“ treatment arm were repleted with 360,000 IU of vitamin D for 12 weeks and then continued with an additional 12 weeks of the same therapy, i.e., with the total dose of 720,000 IU received over 24 weeks. The study protocol (EudraCT 2016-000503-87) was approved by the Central Ethics Committee of the Medical Research Council and registered at clinicaltrials.gov (Clinical-Trials.gov Identifier NCT04840238). The study’s flow chart is provided in Figure 1.

### 2.2. Study Subjects

Patient selection at screening: A diagnosis of trial premenopausal female subjects (age ≥ 18) with PCOS was established based on the revised Rotterdam criteria if two out of the following three features were met: clinical and/or biochemical hyperandrogenism, polycystic ovarian morphology on ultrasound, and/or oligo-/anovulation [47]. These subjects were selected with serum 25-hydroxyvitamin D (25(OH)D) levels between 10 and 30 ng/mL and a BMI < 36. Patients with any signs of hypercalcemia, hypercalciuria, kidney stones, or any chronic disorders that may significantly influence the absorption or metabolism of vitamin D or calcium or with metabolic/endocrine diseases of another origin, or any kind of hormonal or metformin therapy within 6 weeks or vitamin D intake exceeding 1000 IU within the 3 months period prior to screening were excluded.

A total of 177 subjects were screened based on the study criteria between December 2016 and February 2019, and the last follow-up visit was performed in October 2019. There were 115 patients enrolled in the study and randomized into two treatment subgroups. The population of intention to treat (ITT) included all the enrolled patients who met the following criteria:

- Had post-baseline efficacy data regarding cycle length and number of menses cycles and were willing to complete all the study visits and procedures.
- Did not become pregnant during the study.
- Had no signs of other disorders related to the menses cycle (e.g., metrorrhagia).

Accordingly, 31 patients in total were excluded from the statistical analysis due to the reasons of missing cycle data (25 patients), pregnancy (4 patients), and metrorrhagia (1 patient), and 1 patient who had withdrawn her consent.

The participants of the ITT population (n = 84) and each of the treatment arms (D12 and D24) were similar in regard to mean age, BMI, and menses cycle parameters, and no significant differences were detected in the main lab parameters including serum levels of estradiol (E2), testosterone (T), and vitamin D (25(OH)D). None of the following vital parameters showed significant differences between the groups: mean systolic/diastolic blood pressure, pulse rate, and respiratory rate. The potential confounders such as dietary vitamin D intake and physical activity levels were considered at screening. The physical activity levels (total physical activity, PA), and the average weekly workout time as well as the dietary calcium were in the same range; the limitations and vitamin D intake (as 1000 IU/die) were covered in the study exclusion criteria.

### 2.3. Intervention

The enrolled trial subjects were randomly assigned to the two treatment groups. In the study, 30,000 IU vitamin D film-coated tablets (vitamin D_3_ Pharma Patent 30,000 IU film-coated tablets, Pharma Patent Ltd, Budapest, Hungary) or a matching size of placebo tablets in a double-blind manner were administered once a week for 12 weeks which was followed by another 12 weeks of open-label active treatment for both groups with the same dose of 30,000 IU/week. Taken together, all the patients (ITT population) received vitamin D3 continuously from the beginning with a total of 720,000 IU (“D24” group) or only after the 12 weeks of placebo lead-in period with a total of 360,000 IU doses in the “D12” treatment group. This placebo “lead-in period” resulted in a vitamin D-depleted baseline (BL) in order to assess the effect of a consequent active 12 weeks of vitamin D treatment for this group. The other treatment group, “D24”, received 12 weeks of vitamin D in order to achieve the complete repletion of vitamin D levels (repleted baseline), which was then followed by an additional 12 weeks of treatment with the same dose again. To ensure the optimal calcium intake for all the trial subjects who had an average dietary calcium intake below 1000 mg/die, they received a supplementation with 2 × 200 mg tablets with commercially available calcium citrate (Citrokalcium 200 mg tablets, PharmaPatent Ltd., Budapest, Hungary), based on a dietary calcium survey at screening. The treatments were interrupted upon a positive pregnancy test presented at any time of the study period.

### 2.4. Study-Specific Assessments of Ovarian Cycles

The baseline data on the cycle length and regularity based on the dates of the three last cycles, and also the anamnestic data on the frequency/number of menses cycles during the past 6/12 months, as reported by the patient, were recorded. Access to an electronic or paper-based patient diary version was provided to all the study participants to register the dates of menses within the study period and report the use of concomitant medication, as well as the frequency of any other menses cycle-related signs and symptoms, or other complaints. The entries in the patient diaries were reviewed by the study staff during each regular study visit.

### 2.5. Assessment of Ovarian Morphology

A standard transvaginal ultrasonography (TVUS) examination was performed at screening and after each of the study phases at weeks 12 and 24. The ovarian morphology, and the size and number of follicles were recorded via ultrasound imaging. Results which were not more than 3 months prior to screening were acceptable for the study. Ultrasound assessments were categorized into normal ovarian morphology (NOM), polycystic ovarian morphology (PCOM), and unilateral partial remission (pPCO) categories. We also followed the Rotterdam recommendation when providing the sonographic description of PCOM. A patient, based on the TVUS results, was considered as having an “improved” ovarian morphology if the status from PCOM to pPCO or to NOM, or from pPCO to NOM changed. Patients who improved to having a normal morphology were considered as in complete remission. The ratios of the improved patients were compared by applying Chi-square and McNemar tests for both the D12 and D24 group at the end of the double-blind period (at week 12) which were repeated at the end of the open-label vitamin D treatment phase (at week 24).

### 2.6. Laboratory Tests

Blood and urine samples for clinical laboratory assessments were collected at the baseline and during the visits after 12 and 24 weeks in the study. The tests included 25(OH)D, progesterone, 17β-estradiol (E2), FSH, LH, PTH, testosterone (T), androstenedione, sex hormone binding globulin (SHBG), HgbA1c, HCG, and routine lab tests (blood count, biochemistry, ions, and standard urinalysis). The lab tests were performed between days 20 and 25, and progesterone was considered responsive if the value was above a 10 ng/mL cut-off limit. (see also Table 1).

### 2.7. Other/Lifestyle

Specific assessments of daily physical activities and sports were conducted at screening, baseline and throughout the study duration. The investigators asked both open and closed questions to evaluate the regularity (frequency per week), intensity, and duration (0–20–40 or more minutes as an average daily workout). Additionally, they inquired about any changes in regular daily work activities to calculate the average physical activity index.

### 2.8. Statistical Analysis

The primary efficacy endpoint was the ratio of responder patients based on the parameters of ovarian function detected by the elevation in progesterone levels and regularity by using the menses diary. The improvement in ovarian function was assessed by the presence/appearance of elevated progesterone levels in the expected postovulatory phase of the cycle. Changes in hormonal status and 25(OH)D levels were analyzed by using descriptive statistical methods, and these parameters were compared to the baseline values and between the treatment groups.

The ratios of responders through elevated progesterone levels in patients by treatment group were compared by using Pearson’s chi-square test. For the calculation of the 95% confidence interval, the Clopper–Pearson method was applied. We used paired Wilcoxon test for comparisons to the baseline characteristics groups and Mann–Whitney test for the comparisons between groups. The McNemar test was used to determine if there were differences in regard to a dichotomous dependent variable between the two related groups. Additional exploratory efficacy assessment was carried out for subjects with qualitative changes in their ovarian morphology as assessed via TVUS.

## 3. Results

**Figure 2:**
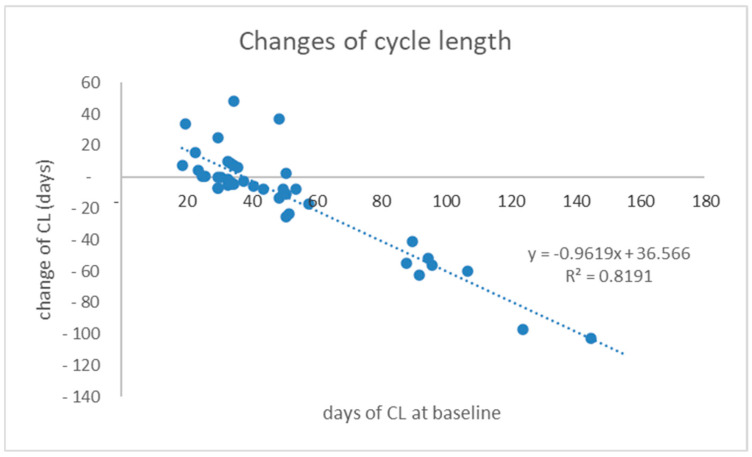
Changes in cycle length (CL) in PCO patients by the end of the 12 weeks of treatment with vitamin D3 (Vit D) and calcium supplements. The horizontal axis presents the cycle length at baseline visit for each individual patient, and the spots represent the +/− change in CL from baseline in treatment group. Linear regression trendline with a negative slope is presented with dotted line.

The treatment arm, “D12”, received supplementation of calcium only during the double-blind placebo lead-in phase. This 12-week “observation” period resulted in non-significant changes in the reported cycle lengths (CLs) and in the study’s specific lab results for testosterone, androstenedione, or the sex hormone binding globulin (SHBG). There was only a minor elevation in group means of 17β-estradiol and 25(OH)D (<5 ng/mL), but no significant change in parathyroid hormone (PTH) or in serum calcium or urine Ca/Cre levels. This placebo “lead-in period” resulted in a vitamin D-depleted baseline (BL) with the mean CL of 51.1 ± 30.29 days (95% CI: 41.66–60.53) (see Table 2). After the 12 weeks of vitamin D3 treatment, a statistically significant decrease in the CL was observed, with a mean change of −11.7 ± 31.9 days (*p* = 0.032). Patients with greater deviations in the CL at the baseline, particularly those with PCOS characterized by more irregular cycles as the oligo-anovulation phenotypes, experienced a pronounced reduction in the CL, as shown in Figure 2. Two-thirds of the patients reported more frequent menses and a reduction in the CL compared to the baseline, with the change averaging −25.6 ± 30.6 days (95% CI: 13.3–38.3). However, among a subgroup of patients with a CL of 35 days or less, which comprised about one-third of the treatment group, CL irregularities remained, and there was a trend towards longer CL periods, averaging +6.35 ± 14.5 days, though this change was not statistically significant (*p* = 0.1005).

**Figure 3:**
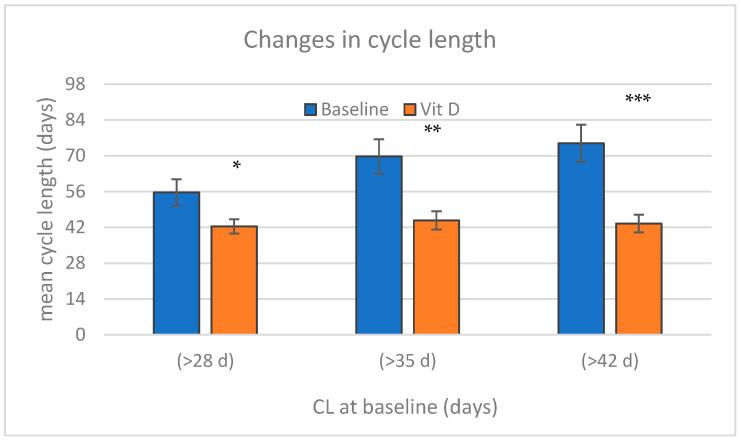
Changes in cycle length (CL) in subgroups of patients with an average length of >28 days, >35 days, or >42 days at baseline as a result of treatment with vitamin D3 (Vit D) and calcium supplements. (Columns represent mean and ±SEM of baseline and at the end of 12 weeks of treatments; asterisks indicate statistical significances of * *p* < 0.05; ** < 0.01; and *** < 0.001).

In order to assess the specific effect of treatment on the regulation of cycles, patient subgroups with CL > 28, CL > 35, and CL > 42 days at the baseline were selected for further analysis, and a comparison of the “treatment-free” depleted BL data with the post-treatment results of vitamin D3 supplementation was performed. (Figure 3) The decrease in the CL was more prominent in patients with initially longer menses cycles (−14.6, −27.9, and −31.4 days, respectively) and statistically significant in each subgroup.

Patients in the “D24” treatment arm were initially loaded with 360,000 IU of vitamin D, and this visit was considered the repleted baseline. The 25(OH)D levels in this group were above 30 ng/mL in most (95%) of the individuals with a mean of over 45 ng/mL. The subsequent 12 weeks of supplementation did not elevate further the mean serum 25(OH)D levels for them. The changes in CL showed a similar range in mean decrease (−10.8 ± 26.3 days) that was observed in the other “D12” treatment group by the end of the treatment period (see also Table 3). Improved regularity and a reduced mean CL were observed in over three-quarters of the patients by the visit at the end of the 24 weeks of the study period; however, no significant changes in mean androstenedione, T, or E2 levels were observed during this subsequent vitamin D supplementation period compared to the values at the repleted baseline visit. Among these responsive patients, the reconciliation of CL was even more prominent within the second part of the 24 weeks at 33.3 ± 7.05 days of reduction (CI: 30.6–35.9).

### 3.1. Serum Androgen and Estrogen Levels

Higher levels of androstenedione (10.6 ± 5.84 nmol/L) were observed at screening in the intention-to-treat (ITT) population. Approximately 40% of the patients were hyperandrogenic, and most of them reported longer cycles with CL > 35 days. This is in agreement with other findings in that the phenotype of androgen excess in PCOS patients is also representing a higher prevalence of vitamin D deficiency [11]. The reconciliation of the lower vitamin D levels and the twelve weeks of vitamin D3 treatment (30,000 IU/week) resulted in a significant increase in serum 25(OH)D levels to 45.1 ± 10.6 ng/mL for the study ITT population (*p* < 0.001). These loading vitamin D doses resulted in a slight decrease but no significant changes in androstenedione or T levels for the ITT population.

**Figure 4:**
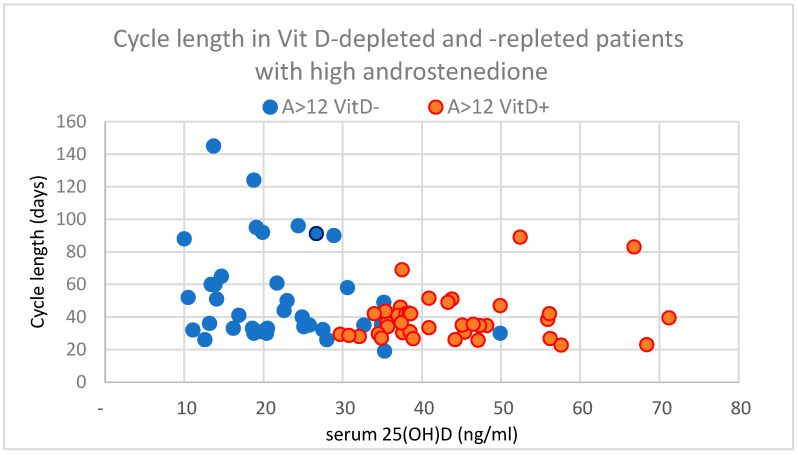
The effect of vitamin D supplementation (360,000 IU) in PCOS (ITT population) in supplemented (VitD+) vs. depleted (VitD−) individual’s cycle length in patients with high androstenedione using the cut-off level of >12 nmol/L.

A post-hoc analysis oof the effect of vitamin D treatment in PCOS patients was performed on the subgroups with high and normal androstenedione levels. For patients with normal androstenedione levels (n = 52; ITT), the majority also exhibited testosterone (T) levels within the normal range (1.7 ± 0.74 nmol/L; CI: 1.49–1.91), and the administration of vitamin D + calcium did not alter the mean T levels. There was a slight increase in the mean estradiol (515.4 ± 375.6 nmol/L; CI: 408–622; *p* = 0.034), but no changes were observed in the CL or other parameters, but the rate of ovulation increased to ≈70% in this group. Conversely, hyperandrogenic patients (n = 32, ITT) with androstenedione levels above 12 nmol/L had initially elevated T levels in more than half of the group, with a mean of 2.56 ± 0.897 nmol/L (CI: 2.32–2.879). Through vitamin D repletion, there was a statistically significant reduction in the mean T levels (*p* = 0.047) and also a decrease (*p* = 0.003) in cycle irregularity to 39.7 ± 15.5 days (see also Figure 4).

A similar reconciliation in mean T levels with a statistically significant decrease (to 2.107 ± 1.052 nmol/L CI:1.64–2.57; *p* = 0.0082) was also found in the subgroup of patients with an LH/FSH ratio greater than 2. In contrast, patients with an LH/FSH ratio less than 2 did not undergo a change in mean testosterone (mean: 1.915 ± 1.00 nmol/L; CI: 1.44–2.39) or a treatment-related change in estradiol levels. For patients characterized with an LH/FSH ratio > 2 and an initial CL > 28 days, the effect of Vit D resulted in T levels being reduced to 2.22 ± 0.98 nmol/L (CI: 1.74–2.71; *p* = 0.014), increased SHBG (*p* = 0.043), and the CL being reduced significantly (*p* = 0.002). Similar trends of T were observed in each subgroup with the CL greater than 35 days (n = 22) and CL greater than 42 days (n = 19), though these changes did not reach statistical significance in our study population.

### 3.2. Assessments of Ovulation

**Figure 5:**
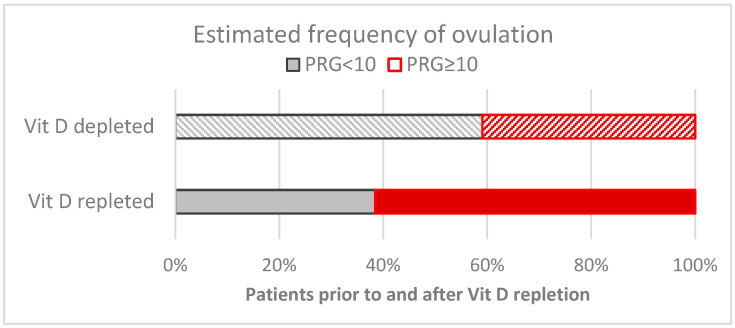
The frequency of ovulation is estimated by the proportion of elevated serum progesterone (>10 nmol/L) among PCOS patients before (Vit D3 depleted) and after (Vit D3 repleted) baseline and after a consequent 12 weeks of treatment with Vit D3 and calcium. The differences were statistically significant. Bars represent the proportion of individuals in a vitamin D-depleted (striped) or -repleted (filled) status.

Progesterone values were determined within the luteal phase (at the baseline visit and repeated by the end of the 12-week-long Vit D treatment). The number of ovulations (estimated by reference to the elevated post-ovulatory progesterone levels using a 10 ng/mL cut-off limit) was ≈40% in untreated patients and increased significantly by the 12th week to 59–65% after vitamin D repletion (κ2: *p* = 0.044, z:2.016). (see also Figure 5). Vitamin D3-repleted patients in the D24 treatment group showed no significant further changes in response to the administration of vitamin D3.

**Figure 6:**
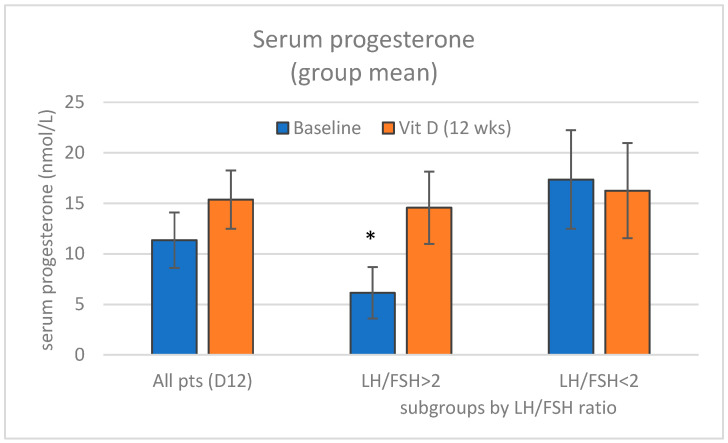
Mean serum progesterone levels among PCOS patients by the end of the treatment with vitamin D_3_ (Vit D3) and calcium. Progesterone levels in patients with an LH/FSH ratio > 2 were significantly lower (* *p* = 0.036) at the baseline.. Columns represent mean ± SEM of serum progesterone for all patients and also the subgroups with LH/FSH ratio > 2 with marked effect and the LH/FSH ratio < 2 subgroup with no meaningful effect of treatment, respectively.

There was a noticeable difference between PCOS patients with a normal and high LH/FSH ratio in relation to the estimated rate of ovulation and mean progesterone levels. Both parameters were higher at the baseline visit among patients with an LH/FSH ratio < 2, but there were no further changes by the end of the 12 weeks of the vitamin D3 treatment period (Figure 6). In contrast, the progesterone levels were significantly lower (*p* = 0.036) at the baseline in patients with an LH/FSH ratio > 2. The treatment with vitamin D3 resulted in a marked increase in the rate of ovulation (≈50% of patients, κ2: *p* = 0.006) in this group and consequently, a statistically significant (*p* = 0.0342) progesterone elevation to a similar level that was observed in patients with an LH/FSH ratio < 2 at the baseline (i.e., vitamin D-depleted patients).

**Figure 7:**
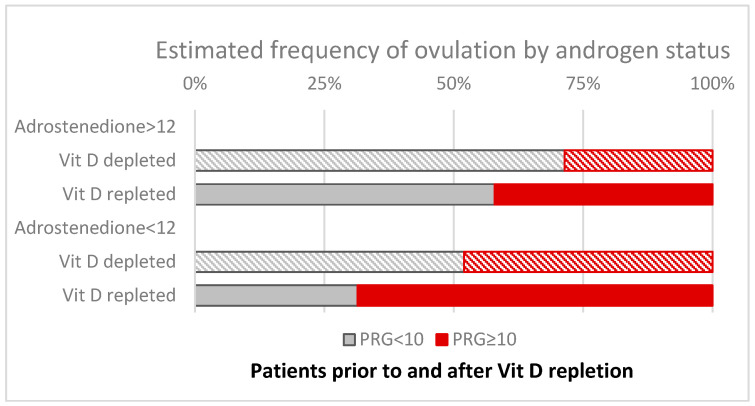
The frequency of ovulation by the stratification of high and normal androstenedione levels (cut-off 12 nmol/L). The rate of ovulation is estimated by reference to progesterone (≥10 nmol/L) in the group of Vit D3-depleted (striped bars) and Vit D3-repleted (filled bars) PCOS patients. The results suggest that higher androgen levels restrict the cycles with ovulation. Bars represent the proportion of individuals by reference to the estimated rate of ovulation.

Vitamin D deficient (depleted) patients with hyperandrogenic phenotypes in PCOS exhibited the most problematic clinical cases since they had the lowest ovulation rate of around 20–30%, and only moderate improvement to approximately 40–45% could be achieved with vitamin D repletion (see also Figure 7) but showed enhancements in cycle regularity (extrapolated, 10 cycles per year).

Vitamin D repletion of 360,000 IU in patients with normal androstenedione resulted in a notable increase in the frequency of ovulation to about 70%, although there was only limited improvement in cycle regularity. The highest ovulation rate, approximately 74%, was observed in PCOS patients with normal androstenedione levels and an LH/FSH ratio of less than 2 in our study population.

### 3.3. Ovarian Morphology

The ovarian morphology, diagnostic signs, and the number of follicles were recorded at each of the study visits. The number of follicles reported via standard TVUS examination showed a trend of a gradual decrease, with no statistical significance. The mean follicle number changed from the initial 29.4 ± 12.6 and 32.7 ± 27.5 to 24.1 ± 13 and 23.8 ± 12.5, respectively, by the end of the trial period in both D12 and D24 treatment groups.

The ratio of patients with “normal” TVUS ovarian images was compared between visits within the treatment groups (ITT population), and the main results are summarized in Table 4. The proportion of trial subjects with a normal morphology showed a statistically significant difference between the placebo and vitamin D3 treatment groups (*p* < 0.05). Twelve weeks of vitamin D3 therapy resulted in normal ovarian status in 20% of the subjects in the study.

Subjects with unilateral changes were considered as having partial improvement in ovarian morphology. Calcium supplementation only applied for 12 weeks during the lead-in period resulted in “partial improvement” in 18.2% of the trial subjects. The subsequent 12 weeks of vitamin D3 treatment further improved ovaries to reach a bilateral normal morphology or unilateral improvement in 38.6% of the patients in group D12. The changes in treatment groups compared to the baseline status were significant (Chi-square test: *p* < 0.05). In treatment group “D24”, the additional 12 weeks of vitamin D3 + calcium resulted in a further increase in the number of normal and partially improved patients (30% to 50%). The total number of improvements, however, was not statistically different between the study groups (Chi-square test at week 24: *p* = 0.6249).

### 3.4. Safety Assessments

No clinically significant deviation in the vital parameters or safety lab results were detected in the study groups. The potential risk of vitamin D overload, as well as early signs of hypercalcemia and hypercalciuria, was investigated as a safety outcome during the combined calcium and/or vitamin D therapy. Serum calcium did not increase significantly as a result of the 12 or 12 + 12 weeks of treatment. The frequency of borderline hypercalcemia (i.e., within 10% of the upper normal limit) observed at the screening visit decreased after 12 weeks of vitamin D therapy, and there were no cases of elevated serum calcium levels after 24 weeks. The risk of hypercalciuria, as assessed by the CA/CRE deviations, remained below 4.5% throughout the loading period, and all were transient in nature. The 12 weeks of 30,000 IU/week loading was effective, as serum 25(OH)D levels increased significantly in all the subjects, with 96% achieving levels of 30 ng/mL. Altogether, 44% of the patients exceeded 45 ng/mL and six (8.3%) subjects were above 60 ng/mL. None of the highest individual 25(OH)D values exceeded 72 ng/mL (180 nmol/L), which is below the safety limit of 85 ng/mL (210 nmol/L). It is noteworthy that none of these elevated 25(OH)D levels were associated with signs of hypercalcemia, and only two cases of transient increases in urinary CA/CRE were detected after loading. No treatment-related serious adverse events (SAEs) were reported during the study. A tabulated summary of the calcium safety data is provided in Appendix A.1.

## 4. Discussion

Our prospective double-blind two-phase randomized controlled clinical trial has demonstrated that vitamin D and calcium supplementation for 12 weeks (without the use of metformin) can improve ovarian morphology, the regularity of menses cycles, and increase the number of ovulations. The improvements in cycle length were more pronounced in patients with an initial CL > 28 days, and a consequent improvement was observed in their oligomenorrhea. This therapeutic potential of vitamin D3 in the management of PCOS opens up new and cost-effective therapeutic approaches to treat this frequent condition.

The application of high-dose vitamin D3 can be beneficial to regulate and normalize the menstrual cycle, as reported by several research groups [10,48,49,50]. A meta-analysis of previous studies suggested that doses of at least 3000 IU/day are required for the adequate supplementation in PCOS [51]; in our study, we used the dose of 30,000 IU/week, which provided sufficient repletion in 12 weeks. The regularity of ovulation was found to be correlated with the vitamin D levels in PCOS [21]. This aligns with experimental results indicating that the vitamin D receptor (VDR) plays a significant role in estrogen production within the ovary. Consequently, vitamin D and calcium are essential for proper gonadal function and the biosynthesis of estradiol, estrone, and progesterone [1,2,40]. Furthermore, vitamin D is involved in regulating the expression of anti-Müllerian hormone (AMH) mRNA in granulosa cells, potentially influencing follicle-stimulating hormone (FSH) sensitivity and thus enhancing follicular maturation in the ovary [6,52]. It was noticed that a smaller subgroup of the participants, without significant abnormalities of ovarian cycles at the baseline (i.e., ovulatory phenotype) failed to show a further reduction in CL. For them, the estimated ovulatory rate was ≈65% similar to that observed in patients with disrupted cycles only after the vitamin D repletion.

In our study, patients with a higher LH/FSH ratio were more responsive to the applied vitamin D dose, indicated by the significant reduction in the cycle length and testosterone levels. The same dose of vitamin D supplementation in patients with an LH/FSH ratio < 2 could not achieve better cycle regularity. Vitamin D3 was suggested to actively influence the pituitary gland in which way the LH/FSH ratio can be more balanced [49]. The study of Lerchbaum et al. (2021) demonstrated a significant negative correlation with 25(OH)D and a higher LH/FSH ratio [53]. The elevated LH/FSH ratio in PCOS patients may indicate the disturbance in the secretion pattern of GnRH due to abnormal negative feedback from ovarian estrogen. Nevertheless, it should be noted that blood sampling was carried out on or after the 20th day of the cycle (i.e., within the luteal phase), since our primary objectives were the detection of ovulation and the assessment of changes in the ovarian morphology. Therefore, this methodology may not be adequate to complete a hormonal profiling and detection of changes in the LH/FSH ratio or E2 secretion patterns.

One of the main characteristics of PCOS (except the phenotype of non-hyperandrogenic PCOS) is an excessive secretion of androgen hormones and the consequent development of hyperandrogenism, which is present in 60–80% of PCOS women [54,55]. This is caused by the disruption of steroidogenesis in the theca cells of the ovaries, thereby increasing the activity of the primary catalyzing enzymes responsible for androgen synthesis. Androgens are considered as functional intermediates in the biosynthesis path of estradiol and also have direct effects on follicular growth in harmony with the LH and FSH stimulatory regulation. It should be noted that hyperandrogenism co-exists not only in the classic PCOS (Phenotype 1) diagnoses but also in the oligo-anovulatory phenotype, as well in phenotypes with evidence of a polycystic ovary [56]. Androstenedione and testosterone are significantly higher among PCOS patients in general compared to otherwise healthy controls; thus, elevated androgens (testosterone and androstenedione) in PCOS patients are considered as the main diagnostic for excess ovarian androgen [55]. There was a higher level of androgens (androstenedione and testosterone) observed in our study population. The loading treatment with vitamin D deficient or insufficient PCOS patients resulted in a significant reduction in testosterone levels and improved cycle regularity among the patients with higher androstenedione levels, but no significant changes in patients with androstenedione in the normal range were detected in our study.

The subgroup analysis also revealed that patients with an LH/FSH ratio greater than 2 demonstrated a substantial reduction in T levels, i.e., responded more effectively to the treatment. In contrast, those with an LH/FSH ratio less than 2 showed little to no improvement in serum testosterone levels. The effect of reduced T levels is in agreement with the previously reported data and also meta-analysis [57] of six clinical trials, in which vitamin D3 and calcium (with metformin) reduced serum testosterone and improved the signs of hirsutism in PCOS patients. Other authors [58] also suggest that adequate circulating vitamin D3 levels may improve PCOS. Our results indicate that vitamin D loading with a total dose of 360,000 IU had a significant impact through reducing the testosterone levels in hyperandrogenic PCOS patients, particularly those with an elevated LH/FSH ratio. This finding underlines the potential importance of the LH/FSH ratio in predicting the treatment response.

Some of the earlier supplementation studies performed in deficient patients (25(OH)D were below 20 ng/mL at the baseline) with similar doses concluded that vitamin D has an impact on reducing higher androstenedione levels with a slight increase in SHBG and non-significant decrease in testosterone [7,18,19,20], but changes in the CL or ovarian morphology were not investigated. A longer treatment duration using a higher total dose of cca. 768,000 IU in the report of Pal et al. showed a moderate decrease demonstrated both in T and androstenedione levels, in a similar range comparable to the effect described with metformin use [59]. In clinical practice, the vitamin D therapies have resulted in a reduction in serum androgens and AMH levels [60]. A recent meta-analysis reported by Avelino et al. described improvements in hirsutism following vitamin D3 supplementation among women with PCOS, indicating reduced total testosterone levels. Sex hormone-binding globulin (SHBG) levels and the Ferriman–Gallwey hirsutism scores also showed beneficial changes. Although these results were not statistically conclusive, they suggest potential positive effects of vitamin D3 on hormonal profiles [61].

PCOS patients with higher androstenedione levels demonstrated a low rate of ovulation. The supplementation of vitamin D among them has only a limited effect through improving the rate of ovulation (i.e., ≈40%), despite the marked effect on testosterone and improved cycle regularity in our study. On the other hand, in non-hyperandrogenic subjects but with an LH/FSH ratio > 2, significant effects associated with vitamin D repletion on the rate of ovulation were detected (≈70%); however, there was no improvement in cycle regularity.

Overall, these results suggest that the treatment was particularly effective for patients with specific PCOS profiles, guiding future therapeutic approaches for managing the hyperandrogenic phenotypes of PCOS patients. Additionally, these patients’ clinical characteristics or laboratory values related to hyperandrogenism also experienced a notable decrease in the CL. It can be concluded that a sufficient level of vitamin D has at least a partial involvement in the normalization of disrupted steroidogenesis, possibly via VDR in ovarian cells that has a role in the steroidogenesis of females via modulating the enzyme activity involved in this process at the granulosa cells of the ovaries. This hypothesis is in agreement that calcitriol may support follicular development, follicle selection, testosterone-induced aromatase activation, and estradiol secretion [17,32,40,41].

Accordingly, the disorders associated with a vitamin D deficiency and the responsiveness to repletion might have an impact on at least two pillars of the regulatory mechanisms controlling the ovarian cycle and the production of ovarian hormones. These various observed responses to Vit D treatment are plausibly explained by the differences between the various phenotypes in PCOS, which are associated with the dysregulation of ovarian steroidogenesis/sensitivity to pituitary trophormones or partially in combination with the dysregulation of adrenocortical steroidogenesis

The advantage of our study is the controlled parallel group design with a placebo lead-in period which is intended to establish the baseline and then investigate the effect of vitamin D3 supplementation in PCOS without the effect of concomitant use of metformin. The impact of androgen levels, the cycle length, and the LH/FSH ratio was investigated. It was demonstrated that, due to the characteristic profile of various PCOS phenotypes, there might be different responses to the applied vitamin D repletion therapy. This underlines the importance of phenotype-differentiated therapeutic approaches to the treatment of PCOS.

The limitations of this trial include the relatively low number of trial subjects as well as the relatively short post-treatment period. The study’s sample size (n = 84 in the intention-to-treat analysis) may limit the generalizability of the findings, but may serve as a pilot result, based on a larger sample size with special attention to the characterization of the phenotypes of PCOS in IN/EX criteria that would enhance the statistical power and broader applicability of the suggested clinical guidelines. The study design was set for 12 + 12 weeks, but an additional 8–12 weeks would have provided added value, especially to detect the changes in the cycle length (CL) or in the driven changes in hormonal regulation.

In order to reduce the effect of potential confounding factors, the dietary vitamin D intake has been limited, and the participants were requested to confirm the intake amount in a self-reported questionnaire at each visit. Similarly, the physical activity levels and the weekly workout time as well as the UVB sun exposure were addressed. Another weak point that is required to be addressed is the schedule of blood sampling for the assessment of ovulation, which is generally based on mid-luteal progesterone levels, but this is fairly difficult in a study population that might have variable cycle durations. On the other hand, serial ultrasound folliculometry can be proposed for the study methodology for future investigations to enhance the study findings and reduce the inter-subjects’ deviations due to blood sampling bias.

## 5. Conclusions

In summary, our results coming from a prospective double-blind two-phase randomized controlled clinical trial corroborate the notion that vitamin D3 treatment might be an independent or adjuvant therapy for PCOS.

## Figures and Tables

**Table 1:** Characteristics of all enrolled patients and the subgroups of “D12” and “D24” at screening.

Population	Dimension	Mean	SD	Min	Median	Max
Study population ITT (n = 84)						
Age	year	27.7	6.2	18	26.5	46
BMI	kg/m^2^	25.2	4.9	17.7	24.5	35.9
Cycle length (average *)	d	48.2	30.3	23	38	180
Cycles/year	#	8.4	3.1	2	9	13
17β-estradiol	pmol/L	319.6	239.0	79.6	221.2	1214
Testosterone	nmol/L	1.96	0.93	0.4	1.9	5.1
Androstenedione	nmol/L	10.6	5.8	1.7	9.7	24.4
25(OH)D	ng/mL	20.0	5.2	10.8	20.7	29.5
Dietary calcium intake	mg/d	684.5	336.2	241	601.5	1892
Treatment arm “D12” group (n = 44)						
Age	year	27.0	6.2	18	25.5	46
BMI	kg/m^2^	24.8	4.9	17.7	23.5	35.9
Cycle length (average *)	d	51.5	36.0	23	40	180
Cycles/year	#	8.3	3.2	2	8.5	13
Estradiol	pmol/L	309.3	233.1	91.3	208.8	1214
Testosterone	nmol/L	2.0	1.1	0.4	2.3	5.1
Androstenedione	nmol/L	11.46	6.36	1.7	10.95	24.4
25(OH)D	ng/mL	20.1	5.1	10.8	20.7	29.5
Dietary calcium intake	mg/d	712.7	294.2	241	689	1178
Treatment arm “D24” group (n = 40)						
Age	year	28.5	6.26	18	28	45
BMI	kg/m^2^	25.5	4.94	17.8	25.5	35.4
Cycle length (average *)	d	44.9	23.22	26	36	150
Cycles/year	#	8.5	2.96	2	9	12
Estradiol	pmol/L	331.3	247.5	79.6	257.3	1074
Testosterone	nmol/L	1.89	0.75	0.7	1.7	3.4
Androstenedione	nmol/L	9.6	5.1	2	9.0	20.3
25(OH)D	ng/mL	19.9	5.4	10.9	20.7	28.2
Dietary calcium intake	mg/d	653.5	378.2	247	562	1892

* Cycle length calculation based on the historical data of the past 6 months prior to screening.

**Table 2:** The main outcome parameters of vitamin D-depleted patients at baseline and after the 12 weeks of vitamin D3 treatment. (n.s.= statistically not significant).

Population Vit D-Depleted Patients	Vit D-Depleted Baseline	Post-Treatment 12 Weeks Vit D	CI Treatment	*p*-Value
Treatment, Group “D12” (n = 44)	Mean ±SD	Mean ±SD	CI 5–95%	
25(OH)D (ng/mL)	24.34 (±9.18)	45.1 (±11.42)	41.63–48.58	<0.0001
Mean cycle length (days)	51.10 (±30.29)	40.42 (±15.98)	35.5–45.3	0.031
Estradiol (pmol/L)	464.4 (±494.6)	474.1 (±477.4)	325.3–622.8	n.s.
Testosterone (nmol/L)	2.16 (±1.016)	2.02 (±1.022)	1.70–2.33	n.s.
Androstenedione (nmol/L)	12.10 (±6.33)	11.77 (±6.134)	9.86–13.68	n.s.
FSH/LH* ratio	2.38 (±1.36)	2.41(±1.36)	2.00–2.83	n.s.

* Blood samples obtained in lutheal phase.

**Table 3:** The main outcome parameters of vitamin D-repleted patients at baseline visit and after 12 weeks of vitamin D3 treatment. (n.s.= statistically not significant).

Population Vit D-Repleted Patients	Vit D-Repleted Baseline	Post-Treatment 12 Weeks Vit D	CI Treatment	*p*-Value
Treatment, Group “D24” (n = 40)	Mean ±SD	Mean ±SD	CI 5–95%	
25(OH)D (ng/mL)	45.07 (±9.89)	43.15 (±8.359]	40.48–45.83	n.s.
Mean cycle length (days)	47.93 (±21.91)	38.75 (±15.03)	33.28–43.03	0.004
Estradiol (pmol/L)	448.8 (±352.9)	445.1 (±281.5)	355.1–535.2	n.s.
Testosterone (nmol/L)	1.89 (±0.653)	1.96 [±0.815)	1.699–2.221	n.s.
Androstenedione (nmol/L)	9.93 (±6.30)	10.23 [±5.38]	8.51–11.95	n.s.
FSH/LH* ratio	2.303 (±1.426)	2.342 (±1.455)	1.877–2.807	n.s.

* Blood samples obtained in lutheal phase.

**Table 4:** Proportion of patients with normal ovarian morphology across study visits assessed via TVUS images (ITT population, D12 and D24 treatment groups).

Visit	Intervention	Normal Ovarian Morphology	D12 Group (n = 44)	D24 Group (n = 40)
Screening	-	Yes	2/44 (4.55%)	1/40 (2.5%)
	-	No	42/44 (95.45%	39/40 (97.5%)
Week 12	Placebo + calcium 12 weeks	Yes	2/44 (4.55%)	-
		No	42/44 (95.45%	-
Week 12	Vit D3 + calcium 12 weeks	Yes	-	6/40 (15%) *
		No	-	34/40 (85%)
Week 24	Vit D3 + calcium 12 weeks	Yes	9/44 (20.45%) **	9/40 (22.5%) ***
		No	35/44 (79.55%)	31/40 (77.5%)

* McNemar test (at week 12): *p* = 0.0625 (ns./borderline); ** McNemar test (at week 24): *p* = 0.0156; *** McNemar test (at week 24): *p* = 0.0078.

## Data Availability

The complete set of research data are available only upon request to Sponsor Pharma Patent Ltd. concerning the “PAT15-PCODD” study due to the data exclusivity of the Sponsor.

## Abbreviations

The following abbreviations are used in this manuscript:

25(OH)D	25-hydroxyvitamin D
AMH	Anti-Müllerian Hormone
BL	baseline
CL	cycle length
E2	17β-estradiol
FSH	Follicle-stimulating hormone
ITT	Intention to Treat
LH	Luteinizing Hormone
NOM	normal ovarian morphology
PA	Physical Activity assessment
PCOM	polycystic ovarian morphology
PCOS	polycystic ovary syndrome
pPCO	unilateral/partial remission of polycystic ovarian morphology
RCT	Randomized Controlled Trials
SHBG	Sex hormone binding globulin
T	Testosterone
TVUS	Transvaginal ultrasonography
UNL	Upper Normal Limit
Vit D	vitamin D3

## Appendix A

### Appendix A.1

**Table A1:** Safety of vitamin D3 loading treatment (30,000 IU/week) in PCOS: serum calcium levels in patients with 25(OH)D > 60 ng/mL. In none of these patients did the serum calcium level exceed the Upper Normal Limit (UNL).

Treatment Duration/Patients	Age (Years)	BMI (kg/m^2^)	Calcium Intake, Dietary (mg/Day)	25(OH)D Baseline (ng/mL)	Se Ca Baseline (mmol/L)	25(OH)D Loaded (ng/mL)	Se Ca Loaded (mmol/L)	CA/CRE Loaded
12 weeks placebo + 12 weeks of Vitamin D administration								
#210	30	26.2	887	25.1	2.49	65.6	2.48	0.13
#211	30.0	28.2	367	26.6	2.55	66.9	2.46	1.28
#310	23.0	17.7	657	15.4	2.44	68.4	2.37	0.10
#617	19.0	29.1	241	22.4	2.40	66.8	2.35	0.28
#807	37.0	19.1	1372	22.6	2.28	70.3	2.27	0.20
#809	30.0	25.6	1089	15.4	2.34	71.2	2.39	0.88
#840	31	21.9	636	27.4	2.32	67.7	2.32	0.15
12 + 12 weeks of Vitamin D administration								
#612	18.0	19.1	541	21.8	2.32	61.7	2.45	0.22

### Appendix A.2

**Table A2:** The assessments of regular average physical activity (aPA) based on self-reported score of frequency, duration, and intensity of weekly indoor/outdoor workouts and the level or absence of regular daily physical activity at work.

Population	Dimension	Mean	±SD	Min	Median	Max
**Study population ITT (n = 84)**						
Weekly workout	minutes	114	90.6	0	90	420
Average regular PA at screening	Score (%)	20.3	16.9	0.0	18.8	80.6
**Treatment arm “D12” group (n = 44)**						
Weekly workout at the depleted baseline	minutes	95.5	77.9	0	120	300
Weekly workout post-treatment	minutes	85.5	72.7	0	80	240
Average regular PA at the depleted baseline	Score (%)	15.8	13.8	0.0	12.9	48.4
Average regular PA post-treatment	Score (%)	14.4	13.3	0.0	12.9	54.8
**Treatment arm “D24” group (n = 40)**						
Weekly workout at the repleted baseline	minutes	113	80.5	0	120	360
Weekly workout post-treatment	minutes	119	90	0	120	300
Average regular PA at the repleted baseline	Score (%)	22.0	16.6	0.0	19.4	80.6
Average regular PA post-treatment	Score (%)	22.2	14.8	0.0	20.4	80.6
